# Increased Granulocyte Heparanase Activity in Neutrophils from Patients with Lupus Nephritis and Idiopathic Membranous Nephropathy

**DOI:** 10.1007/s00005-016-0396-8

**Published:** 2016-04-18

**Authors:** Maciej Szymczak, Jakub Kuźniar, Wacław Kopeć, Marcelina Żabińska, Zofia Marchewka, Katarzyna Kościelska-Kasprzak, Marian Klinger

**Affiliations:** 1 Department of Nephrology and Transplantation Medicine, Wroclaw Medical University, Wroclaw, Poland; 2 Department of Toxicology, Wroclaw Medical University, Wroclaw, Poland

**Keywords:** Heparanase, Granulocytes, Glomerular diseases

## Abstract

Heparanase is a β-glucuronidase that cleaves sugar chains of heparan sulfate proteoglycans. It is believed that heparanase may be involved in the pathogenesis of proteinuria. The aim of this study was to assess the significance of heparanase in the pathogenesis of particular glomerulonephritis types. The evaluation of heparanase activity in serum, urine, and granulocytes and superoxide dismutase (SOD) activity in granulocytes of patients with lupus nephritis (*n* = 17), membranous nephropathy (*n* = 11), IgA nephropathy (*n* = 12), focal and segmental glomerulosclerosis (*n* = 18), mesangiocapillary glomerulonephritis (*n* = 12) and in 19 healthy volunteers were performed. The heparanase activity in granulocytes of patients with lupus nephritis and membranous nephropathy was higher than heparanase activity in granulocytes in the control group (*p* = 0.02 in both cases). This is the first observation of this phenomenon. There was no difference between SOD activity in granulocytes of patients with all assessed types of glomerulonephritis and the control group. A positive correlation between heparanase activity in urine and double-strain DNA antibodies (*r* = 0.51; *p* = 0.04), and reverse correlations between heparanase in urine and hemolytic activity of the complement (*r* = −0.57; *p* = 0.03) in the lupus nephritis group, and between heparanase activity in granulocytes and serum total protein level (*r* = −0.69; *p* = 0.02) in membranous nephropathy were observed. Increase in heparanase activity without changes in superoxide dismutase activity in the granulocytes from patients with lupus nephritis and membranous nephropathy was observed. It may be used as one of the markers of these disease activities.

## Introduction

Heparanase is an endo-β-glucuronidase that cleaves heparan sulfate (HS) chains connected with heparan sulfate proteoglycan (HSPG) core protein (Nakajima et al. 1984). It had been supposed that pathological amounts of heparanase in the kidney glomeruli might cause proteinuria by the digestion of HSPGs in the glomerular basement membrane (GBM) (Levidiotis et al. 2004), but later data did not support this thesis (van den Hoven et al. 2008).

Heparanase may also act as a signaling and cell adhesion molecule in physiological pH without cleaving HSPGs (Goldshmidt et al. 2003).

Increase in heparanase expression in the kidney glomeruli was observed in the course of diabetic nephropathy (Katz et al. 2002), passive Heymann nephritis (Levidiotis et al. 2004), puromycin (Levidiotis et al. 2001), and adriamycin nephropathy (Kramer et al. 2006), steroid-sensitive nephrotic syndrome in children (Holt et al. 2005), focal and segmental glomerulosclerosis, minimal change disease, IgA nephropathy (van den Hoven et al. 2007), anti-GBM antibody-related disease (Levidiotis et al. 2005), and amyloidosis in humans (Li et al. 2005).

The data on urinary and serum heparanase in adult nephrotic syndrome are ambiguous. There are data suggesting that patients with adult nephrotic syndrome have decreased heparanase concentration in the urine and serum (compared with healthy controls) (Holt et al. 2005). Other authors indicate that urinary heparanase excretion is increased in the course of membranous glomerulopathy, focal, and segmental glomerulosclerosis, and minimal change disease (van den Hoven et al. 2007). It is also known that primed granulocytes can produce heparanase (Cohen-Mazor et al. 2008). Granulocyte priming occurs in the course of chronic kidney disease (Sela et al. 2005). The significance of heparanase produced by primed granulocytes in the course of particular glomerular diseases is still unknown.

This study was designed to assess the heparanase activity in serum, urine, and granulocytes from patients with specific kinds of glomerulopathy. Moreover, superoxide dismutase (SOD) activity in granulocytes was evaluated, to assess the intensity and specificity of granulocyte priming in particular types of glomerulonephritis.

## Materials and methods

The evaluation of heparanase activity in serum, urine, and granulocytes and SOD activity in granulocytes of patients with lupus nephritis (*n* = 17; 11 patients with class IV lupus nephritis, three patients with class III lupus nephritis, one patient with class V lupus nephritis, two patients with non-specified class lupus nephritis), membranous nephropathy (*n* = 11), IgA nephropathy (*n* = 12), focal and segmental glomerulosclerosis (*n* = 18), mesangiocapillary glomerulonephritis (*n* = 12) and in 19 healthy volunteers were performed. All patients signed informed consent for taking part in the study. There was no special key to choose the patients. They were consecutive patients who went to our clinic. The study was performed without any special randomization. The inclusion criteria were known histopathologic diagnosis and proteinuria present in the last accessible test sample of urine. Exclusion criteria were malignancy (diagnosed in the past or treated in the present), advanced chronic kidney disease (creatinine level more than 3 mg/dl), and active infections at the moment of material collection.

The control group consisted of 19 healthy volunteers who signed informed consent. All persons in the control group have serum creatinine within the normal range and lack of proteinuria in urinalysis.

Blood collection was performed using additional tubes during the standard blood sampling for laboratory procedures. The blood was sampled from forearm veins. The blood was collected into tubes with ethylenediaminetetraacetic acid (EDTA) as an anticoagulant (Shafat et al. 2006). For heparanase assessment in serum, 5.4 ml of blood was collected. For heparanase and dismutase assessment in granulocytes, 12 ml of blood was collected (both samples derived from the same vein puncture, but from separate tubes). Blood was collected between 8 and 9 AM. Patients were not obliged to omit breakfast. To take into account the influence of glucose on heparanase, the glucose level was also assessed. All the patients had a glucose level no higher than 140 mg/dl. The blood was transported to the laboratory within 10 min of sampling (heparanase *T*½ = 20 min at pH 7.4) (Ihrcke et al. 1998). The clinic and laboratory are very close to each other so that was possible to transport material very fast (less than 10 min). The time of transport was checked. To be sure that heparanase activity will be diminished in the same ratio, in all patients the centrifugation was started after the same time from sampling (10 min) in all cases. The material was not collected when temperature was higher than 28 °C. Next, blood was centrifuged at 1500×*g* for 10 min at room temperature. After that, obtained serum was frozen at –80 °C. For assessment of heparanase and dismutase in granulocytes (the enzymes within granulocytes are more stable—heparanase is stable for about 16 h) (Nadav et al. 2002), granulocytes were isolated on Ficoll and then frozen at −80 °C. Urine (50 ml) was collected into plastic containers for urine. Urine was collected in the morning from the second portion after awakening. The pH of urine was checked. Heparanase is most stable in pH 5.5 (much more than in pH 7.4). It was found that inactivation of heparanase is common in pH 7.4, but not in pH less than 7. Moreover, inactivation of heparanase is reversible after pH decrease (Ihrcke et al. 1998). To exclude the influence of pH, only urine with the pH 5–6.5 was collected. In case that pH of urine was more than 6.5, material was taken from the patient another day (when pH was 5–6.5). Urine was centrifuged at 1500×*g* for 10 min, and then, the obtained supernatant was frozen at −80 °C.

### Laboratory Methods: Evaluation of Enzymes

#### Heparanase Assessment

Heparanase activity was assessed using an AMS Biotechnology (Europe) Kit.

*Principle of the test* Biotinylated HS is embedded in 96 wells of a polystyrene plate. Heparanase partly degrades HS to fragments that are removed by fourfold flushing with phosphate-buffered saline (PBS)/Tween-20. Heparan sulfate that is remaining in wells binds with heparanase labeled with streptavidin. Substrate in the presence of the heparanase gains a color with a different optical density (OD) from the control OD without heparanase. Optical density of the mixture reactive in the presence of the heparanase divided by control OD is proportional to heparanase activity in the assessed samples.

Heparanase activity is calculated from the formula:

$${\text{R}} = \left( {\left( {\text{OD}} \right)/\left( {\text{MaxOD}} \right)} \right) \times 500$$

where MaxOD is maximal value in the control samples, OD is value in the evaluated samples.

The result is in ng HS released within 1 min as a result of heparanase action. Specific activity is calculated in ng HS per mg of protein. Heparanase activity was assessed in serum, urine, and granulocytes.

#### Superoxide Dismutase Assessment

Assessment was performed using the Superoxide Dismutase Assay Kit (Cayman Chemical Company, Elisworth Rd., Ann Arbor). This kit contains tetrazolium salts O_2_^−^ produced by xanthine oxidase and hypoxanthine. One unit of SOD activity is the amount of the enzyme necessary to inhibit 50 % of O_2_^−^ dismutation. Combined SOD activity (Cu/Zn SOD, Mn SOD, Fe SOD) was assessed.

#### Isolation of Granulocytes from Peripheral Blood

Granulocytes were isolated from 10 to 12 ml of fresh blood anticoagulated using EDTA according to the modification of the Boyum method (Boyum 1968) on Ficoll-Paque. Four parts of twice diluted blood (PBS) were piled up on three parts of gradient Ficoll-Hypaque and centrifuged (300×*g*) room temperature for 30 min. In the layer over the gradient, mononuclear cells are present, and below the gradient samples contain erythrocytes and granulocytes. Granulocytes were isolated from that layer according to the modified method of Baron and Ahmed (1969). Dextran solution (mol mass 250 kDa, 6 % weight/vol–weight divided by volume) in isotonic buffer Tc 199 was added to the blood (layer on the bottom of the sample)—in the proportion one part dextran and four parts blood (vol/vol–volumetric ratio). Next, samples were incubated at room temperature in the vertical position for 30 min for erythrocyte sedimentation. Fluid over the precipitate was centrifuged (300×*g*). Erythrocytes present in the precipitate were lysed using deionized water. The mixture was brought to isotonicity by adding a fourfold amount of isotonic buffer Tc 199. Isolated granulocytes were suspended in PBS solution at pH 7.4. Vitality of granulocytes was assessed with trypan blue and was more than 90 %. Clarity of the granulocyte suspension was 98 %. Isolated granulocytes were centrifuged at 400×*g* for 5 min and then suspended in HEPES buffer/glucose with addition of 0.2 % vol/vol/Triton X-100 and frozen at −80 °C. After defrosting, granulocytes were lysed using the Qproteome Cell Compartment Kit (Qiagen, Hilden, Germany). The suspension contained debris of granulocytes. Heparanase and dismutase were assessed in the fluid over the precipitate with addition of aprotinin 125,000 IU/ml. Proteins were also assessed in that fluid using the Lowry method (microadaptation of Lowry method) (Lowry et al. 1951).

### Statistical Methods

#### Quantitative Variables

Obtained data were analyzed with application of correlation analysis. Most data do not have a normal distribution (Anderson–Darling test). Spearman’s rank correlation coefficient was applied to analyze data in the case of non-normal distribution in both specimens, and Pearson’s correlation coefficient was applied when at least one specimen had a normal distribution in the case of quantitative variables. After that, results were tested in terms of statistical significance with the *t* test for the Spearman and Pearson correlation coefficients. In all conducted statistical analyses, associations with *p* < 0.05 were considered statistically significant. The associations between the following variables were tested: heparanase activity in serum, urine, and granulocytes, dismutase activity in granulocytes, and clinical and laboratory parameters of patients: quantitative—age (years), weight (kg), blood pressure (mmHg), proteinuria (mg/dl), creatinine in urine (mg/dl), leukocyturia (number of leukocytes/µl), erythrocyturia (number of erythrocytes/µl), pH of urine, total protein level in serum (g/dl), albumins in serum (g/dl), creatinine in serum (mg/dl), disease duration (days), total cholesterol in serum (mg/dl), high-density lipoprotein cholesterol in serum (mg/dl), low-density lipoprotein cholesterol in serum (mg/dl), triglycerides in serum (mg/dl), antinuclear antibodies in serum (dilution titer when antibodies were still detected), double-strain deoxyribonucleic acid (dsDNA) antibodies in serum (IU/ml), complement in serum (CH50 units—amount of complement which causes lysis of 50 % of erythrocytes in suspension), C-reactive protein in serum (mg/dl), activated partial thromboplastin time in serum (seconds), leukocytosis in peripheral blood (number of leukocytes/μl), glucose in serum (mg/dl), hemoglobin concentration in serum (g/dl), percent of glomeruli with proliferation, percent of glomeruli with sclerosis, and renal score of Systemic Lupus Erythematosus Disease Activity Index (SLEDAI). All clinical parameters derive from the period when the material for heparanase in serum, urine, and granulocytes and dismutase in granulocytes was collected.

#### Qualitative Data

Data with a normal distribution were analyzed with Student’s *t* test (for two categories) or analysis of variance (ANOVA) (for more than two categories). Data with a non-normal distribution were analyzed with the nonparametric Mann–Whitney test (for two categories) or Kruskal–Wallis test (for more than two categories). The associations between the following results were assessed: heparanase activity in serum, urine, and granulocytes, SOD in granulocytes, presence in kidney biopsy specimens of deposits of IgA, IgG, IgM, C3 (complement component), Ig lambda, proliferation, hyalinosis, thickening of basement membranes in glomeruli, percentage of glomeruli with capsular fibrosis, presence of crescents, necrosis of vascular loops, and tubulointerstitial fibrosis.

### Comparison of Control Group with Study Group

Heparanase in serum had a normal distribution. Analysis of these variables was performed using the ANOVA method and Tukey test. Other data had a non-normal distribution, and then, the Kruskal–Wallis test and Tukey test were applied. The variable sex was assessed with the chi-square test.

## Results

There were no statistically significant differences between the control group and the other groups in terms of age (Holt et al. 2005), sex, or glucose level (Maxhimer et al. 2005), which might be factors that influence the results. In the control group, women have higher heparanase levels in serum than men (*p* < 0.05). Median creatinine level was lower in the control group (1.1 mg/dl) than in patients with lupus nephritis (1.2 mg/dl), membranous nephropathy (1.29 mg/dl), focal and segmental glomerulosclerosis (FSGS; 1.24 mg/dl), or mesangiocapillary glomerulonephritis (1.29 mg/dl) (*p* < 0.05). Median creatinine from the patients with IgA nephropathy was 1 mg/dl.

Clinical Data of the Patients are Presented in Enclosed Table (Table 1).

Median values of heparanase in serum, urine, and granulocytes (ng HS/min/mg of protein), and dismutase in granulocytes (U/ml) assessment in particular types of glomerulonephritis are presented in Table 2.

**Table 1:** Clinical data of the patients

	Control group	Lupus nephritis	Membranous nephropathy	IgA nephropathy	FSGS	Mesangiocapillary glomerulonephritis
Median creatinine level (mg/dl)	1.1 Range 0.71–1.3	1.2 Range 0.75–2.38	1.29 Range 1.11–2.89	1 Range 0.77–2.58	1.18 Range 0.76–2.32	1.29 Range 0.8–2.91
Median eGFR MDRD (ml/min/1.73 m^2^)	71 Range 61–104	56 Range 26–99	51 Range 24–69	78 Range 25–121	62 Range 31–87	57 Range 21–94
Median proteinuria (mg/g creatinine)	0	150 Range 25–968	289 Range 75–1667	150 Range 25–1124	178 Range 25–946	324 Range 25–1224

*eGFR MDRD* estimated glomerular filtration rate calculated using the modification of diet in renal disease formula

**Table 2:** Results of heparanase in serum, urine and granulocytes (ng HS/min/mg of protein), and dismutase in granulocytes (U/ml) assessment in particular types of glomerulonephritis (median values)

	Lupus nephritis (*n* = 17)	Lupus nephritis class IV (*n* = 11)	Lupus nephritis class V (*n* = 1)	Lupus nephritis class III (*n* = 3)	Membranous nephropathy	IgA nephropathy	FSGS	Mesangiocapillary glomerulonephritis	Control
Heparanase in granulocytes	2.39 Range 1.23–4.21	2.39 Range 1.23–3.81	4.21	2.35 Range 1.95–2.78	2.24 Range 0.9–4.05	1.48 Range 0.85–2.8	1.65 Range 0.51–4	1.43 Range 0.9–3.58	1.30 Range 0.4–2.3
Heparanase in serum	2.08 Range 1.08–5.1	1.68 Range 1.08–5.1	2.89	2.48 Range 1.58–4.6	2.66 Range 1.16–4.4	2.64 Range 1.17–5.32	3 Range 1.2–4.88	2.38 Range 1.2–3.56	2.91 Range 1.25–3.9
Heparanase in urine	2.24 Range 1–5.8	2.24 Range 1–5.8	4.9	1.72 Range 1.37–3.52	1.54 Range 0.87–3.4	1.86 Range 1–3.89	2 Range 0.54–5.07	1.87 Range 0.8–4	1.52 Range 0.84–4
Superoxide dismutase in granulocytes	0.42 Range 0.09–1.2	0.42 Range 0.17–1.2	0.92	0.26 Range 0.09–0.5	0.32 Range 0.08–1.16	0.28 Range 0.07–1.04	0.4 Range 0.15–1.48	0.47 Range 0.12–1.2	0.36 Range 0.05–0.83

### Heparanase Activity in Serum

There was no difference between heparanase activity in serum of patients with particular types of glomerulonephritis and the control group.

### Heparanase Activity in Urine

**Fig. 1:**
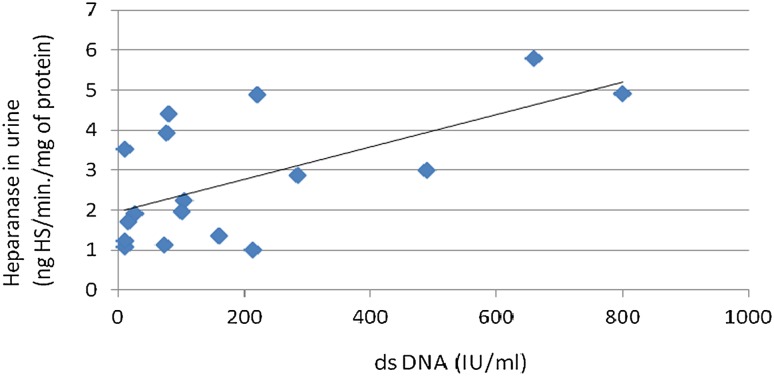
Association between heparanase in urine and ds DNA in patients with lupus nephritis

**Fig. 2:**
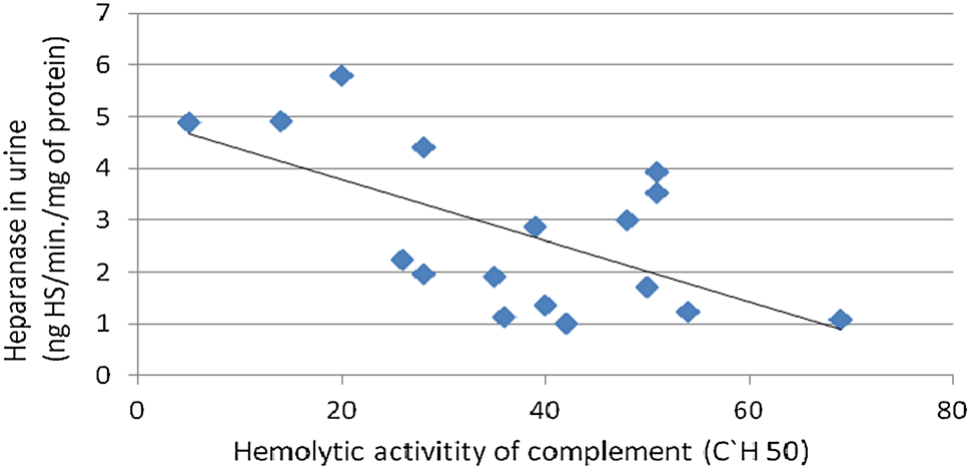
Association between heparanase in urine and hemolytic activity of the complement in patients with lupus nephritis

There was no difference between heparanase activity in urine of patients with specific types of glomerulonephritis and the control group. Correlations between heparanase activity in urine and dsDNA antibodies (*r* = −0.51; *p* = 0.04; Fig. 1), and between heparanase in urine and hemolytic activity of the complement (*r* = −0.57; *p* = 0.03; Fig. 2) in the lupus nephritis group were found. Despite correlations with lupus nephritis markers, there was no significant correlation between heparanase activity in urine and renal score of SLEDAI.

Although the heparanase activity was not significantly higher in the urine of patients with FSGS, the heparanase in urine was correlated with heparanase in serum (*r* = 0.7; *p* = 0.001), heparanase in granulocytes (*r* = 0.69; *p* = 0.006), and dismutase in granulocytes (*r* = 0.6; *p* = 0.02), in this group of patients. Moreover, the heparanase levels in urine were negatively correlated with the time from the beginning of the disease (*r* = −0.66; *p* = 0.003).

### Heparanase Activity in Granulocytes

**Fig. 3:**
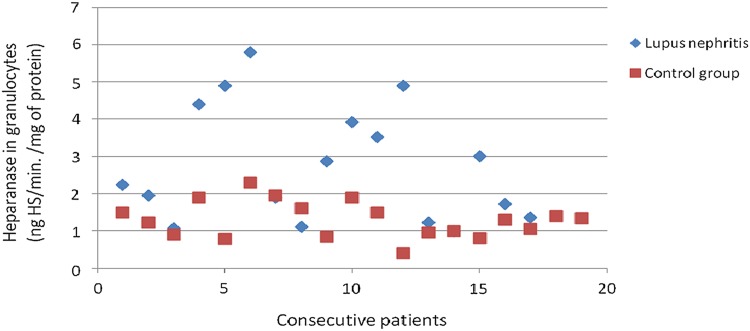
Heparanase in granulocytes of patients with lupus nephritis

The heparanase activity in granulocytes of patients with lupus nephritis was higher than heparanase activity in granulocytes in the control group (*p* = 0.02; Fig. 3). The heparanase activity in granulocytes of patients with lupus nephritis class IV was also higher than heparanase activity in granulocytes in the control group (*p* < 0.05). There was no significant correlation between heparanase activity in granulocytes and SLEDAI renal score.

**Fig. 4:**
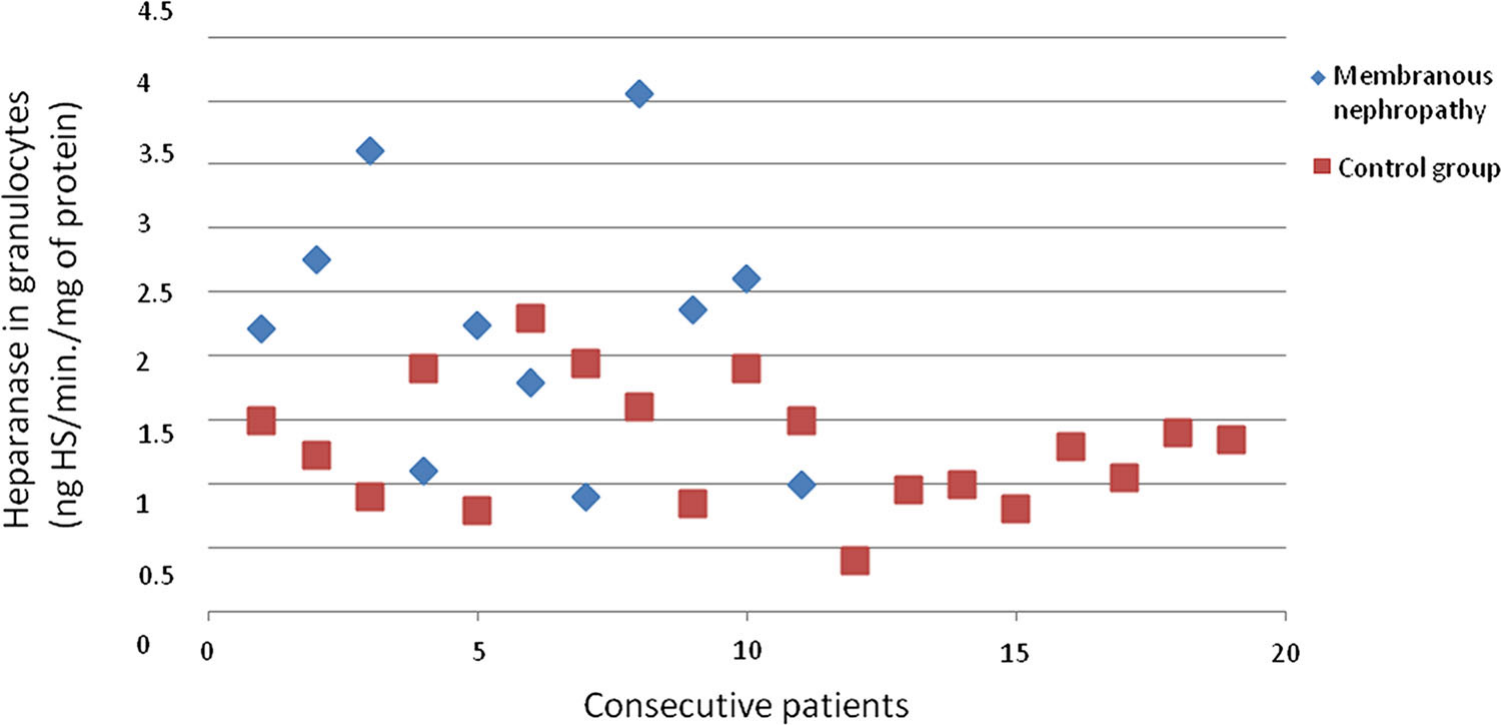
Heparanase in granulocytes of patients with membranous nephropathy

**Fig. 5:**
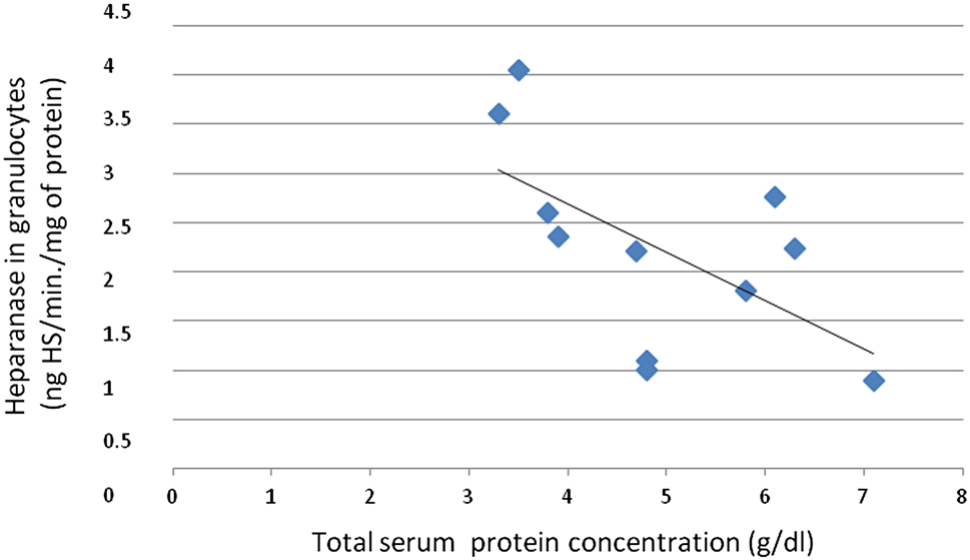
Association between heparanase in granulocytes and total serum protein concentration in patients with membranous nephropathy

The heparanase activity in granulocytes of patients with membranous nephropathy was also higher than heparanase activity in granulocytes in the control group (*p* = 0.02; Fig. 4). A correlation between heparanase activity in granulocytes and serum total protein level (*r* = −0.69; *p* = 0.02; Fig. 5) in membranous nephropathy was observed.

### Superoxide Dismutase Activity in Granulocytes

**Fig. 6:**
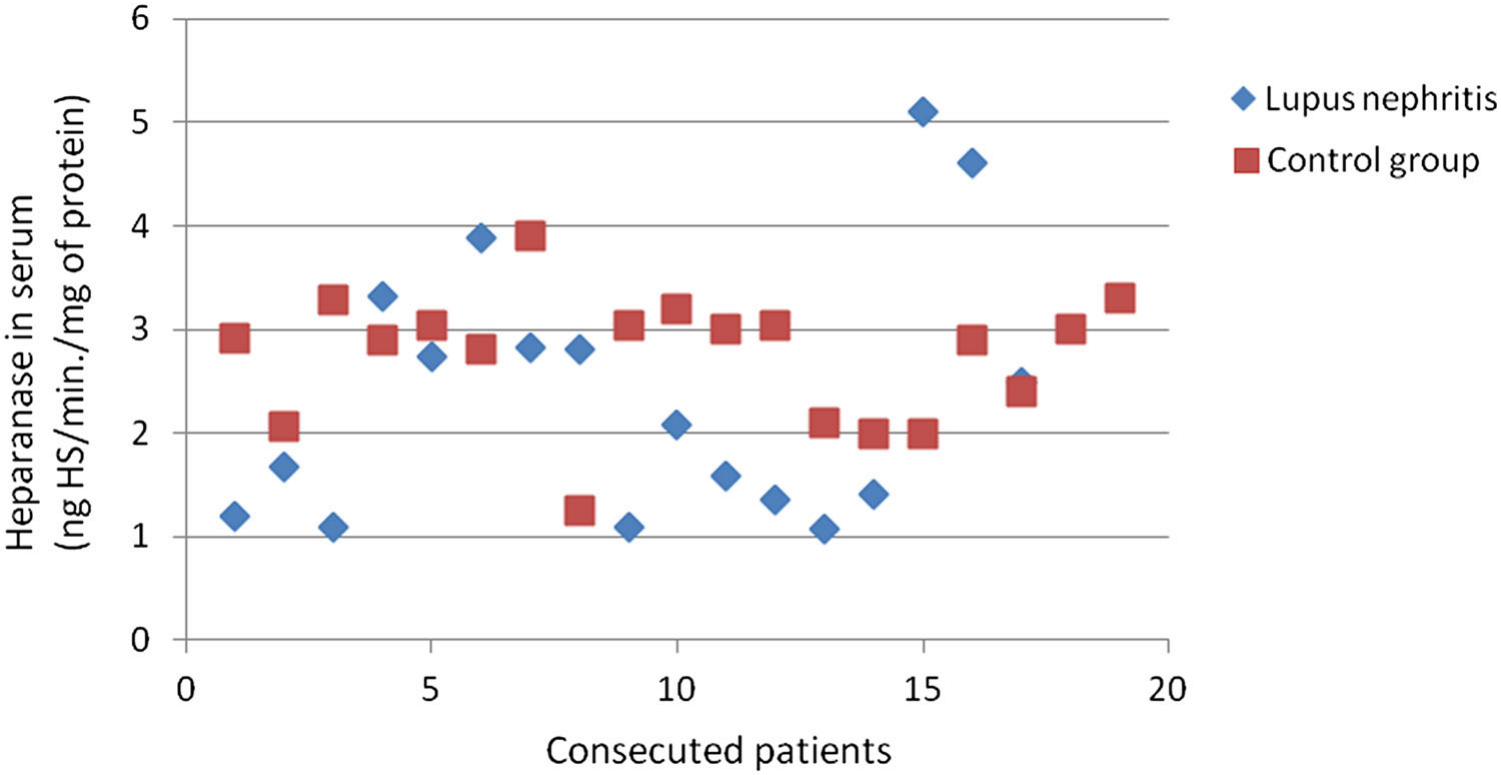
Heparanase in serum of patients with lupus nephritis

**Fig. 7:**
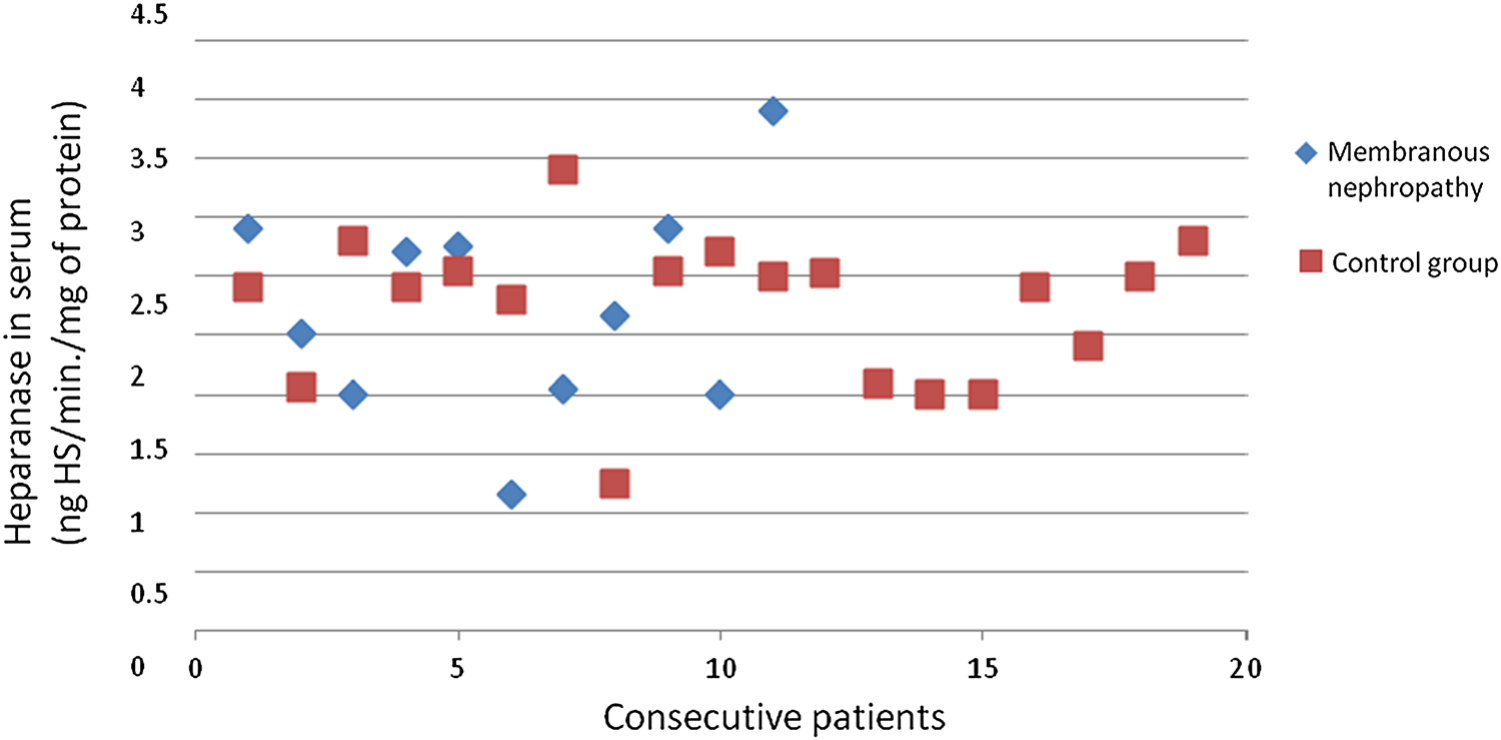
Heparanase in serum of patients with membranous nephropathy

**Fig. 8:**
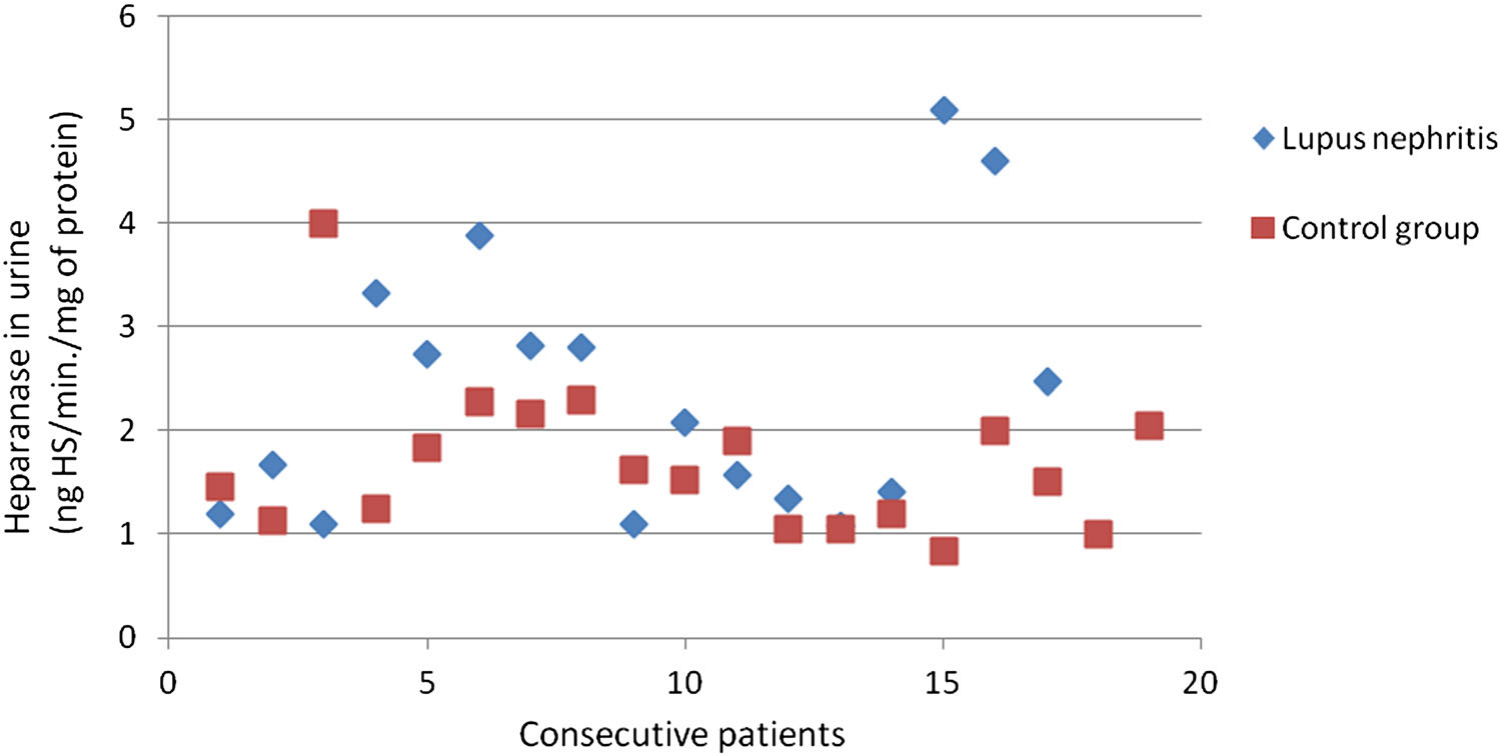
Heparanase in urine of patients with lupus nephritis

**Fig. 9:**
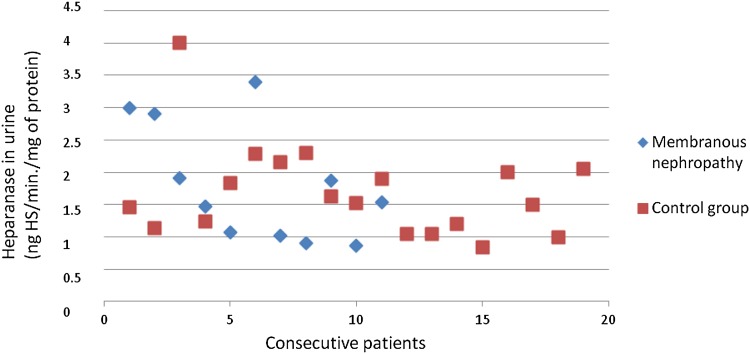
Heparanase in urine of patients with membranous nephropathy

**Fig. 10:**
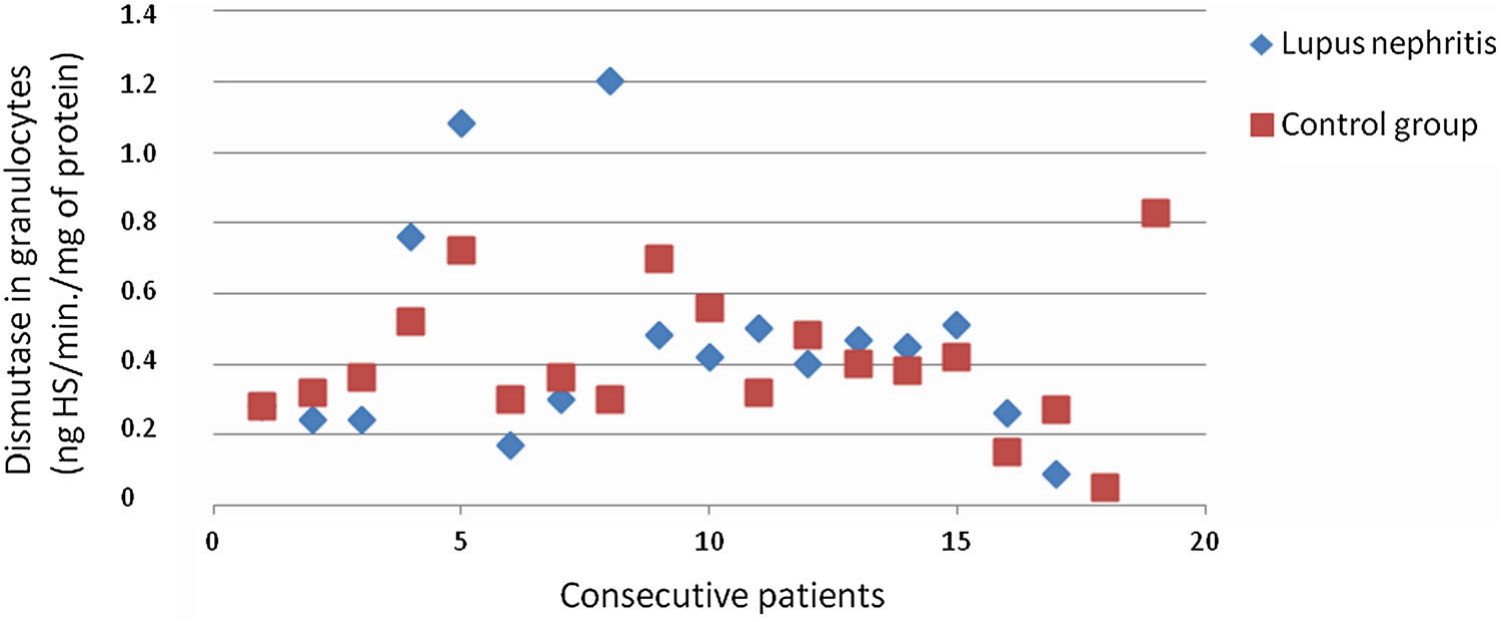
Dismutase in granulocytes of patients with lupus nephritis

**Fig. 11:**
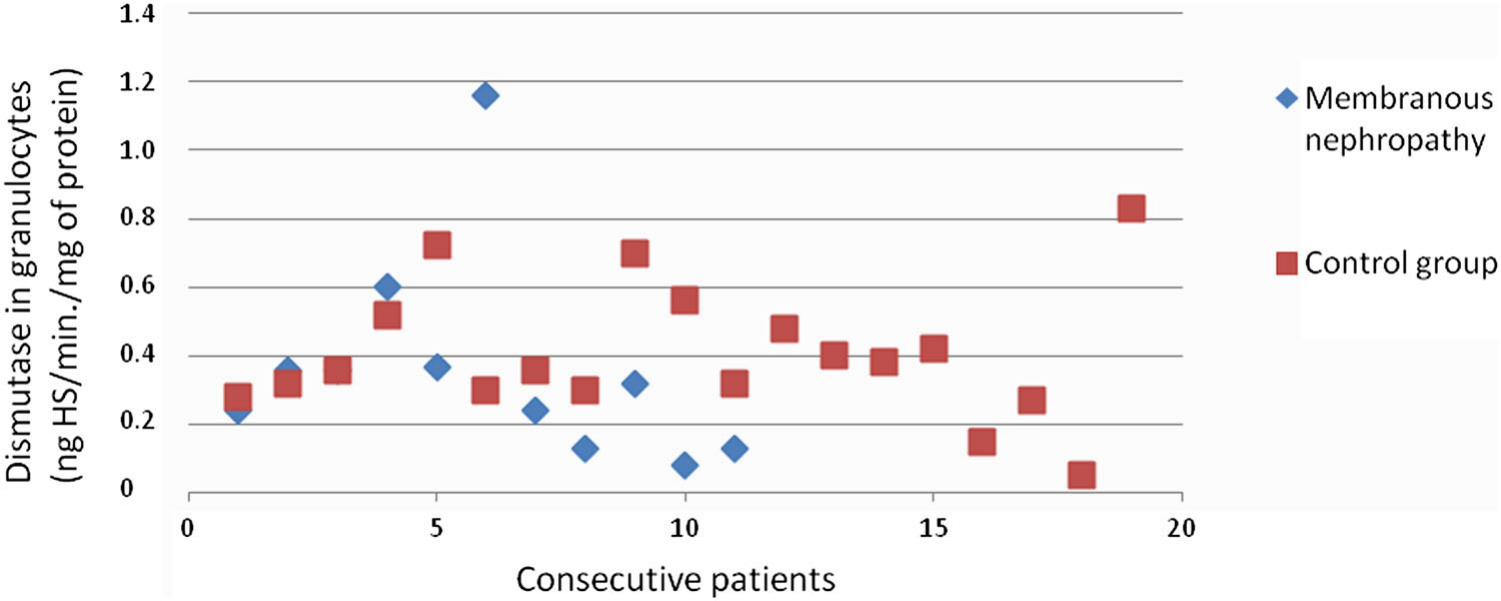
Dismutase in granulocytes of patients with membranous nephropathy

There was no difference between SOD activity in granulocytes of patients with all assessed types of glomerulonephritis and the control group. Figures 3, 4, 6, 7, 8, 9, 10, 11 present heparanase in granulocytes, serum, urine (ng HS/min/mg of protein), and dismutase in granulocytes (U/ml) assessment in particular patients with lupus nephritis and membranous nephropathy.

## Discussion

The results of this study show the increase in heparanase activity in the granulocytes from patients with lupus nephritis and membranous nephropathy. This elevation of heparanase activity was a distinctive event not accompanied by general features of granulocyte activation reflected by normal dismutase activity. It is the first observation of this phenomenon. Existing data indicated exclusively that hemodialysis patients have higher serum heparanase levels and higher heparanase levels in granulocytes, but such an increase is connected with severely deteriorated renal function (Cohen-Mazor et al. 2008). There is also evidence indicating that in chronic kidney disease, granulocytes are primed, but all available data about this fact involved patients with advanced chronic kidney disease and heparanase was not checked in these evaluations (Sela et al. 2005). Lack of superoxide dismutase activity increase indicates that there is no proof that granulocytes from patients in this study were primed in classical way.

The connections of the urine heparanase activity with dsDNA antibodies and hemolytic activity of complement suggest that heparanase may be a useful indicator of lupus nephritis immunological activity. These observations are consistent with previous observations where the loss of HSPGs in the course of lupus nephritis and membranous nephropathy was observed (van den Born et al. 1993).

The observation that heparanase in granulocytes of the patients with membranous nephropathy is increased in comparison with healthy controls is enhanced by the negative correlation between granulocyte heparanase and total protein serum level. Both these observations suggest the thesis that heparanase may play a role in membranous glomerulopathy pathogenesis and may serve as a marker of membranous glomerulopathy activity.

The inverse correlation between heparanase in urine activity in the course of the FSGS and the time from the disease onset suggests that heparanase may be significant for the FSGS course, especially at the beginning of this disease. This thesis is consistent with the observations of the other authors derived from animal models of this disease, suggesting the increase in heparanase during the first days after induction of the disease with constant heparanase expression later (Kramer et al. 2006; Levidiotis et al. 2001). To evaluate fully the role of heparanase in glomerular kidney diseases, further investigations involving larger groups of patients and other techniques, for example, mRNA heparanase expression evaluation are required.

## Abbreviations

CH50 units: Amount of complement which causes lysis of 50 % of erythrocytes in suspension
dsDNA: Double-strain deoxyribonucleic acid antibodies
EDTA: Ethylenediaminetetraacetic acid
FSGS: Focal and segmental glomerulosclerosis
GBM: Glomerular basement membrane
HS: Heparan sulfate
HSPGs: Heparan sulfate proteoglycans
IU: International units
OD: Optical density
PBS: Phosphate-buffered saline
SLEDAI: Systemic lupus erythematosus disease activity index
SOD: Superoxide dismutase
Vol: Volume
